# The impact of education on risk factors and the occurrence of multimorbidity in the EPIC-Heidelberg cohort

**DOI:** 10.1186/1471-2458-8-384

**Published:** 2008-11-11

**Authors:** Gabriele Nagel, Richard Peter, Stefanie Braig, Silke Hermann, Sabine Rohrmann, Jakob Linseisen

**Affiliations:** 1 Institute of Epidemiology, Ulm University, Ulm, Germany; 2 Division of Cancer Epidemiology, German Cancer Research Centre, Heidelberg, Germany

## Abstract

**Background:**

In aging populations, the prevalence of multimorbidity is high, and the role of socioeconomic status and its correlates is not well described. Thus, we investigated the association between educational attainment and multimorbidity in a prospective cohort study, taking also into account intermediate factors that could explain such associations.

**Methods:**

We included 13,781 participants of the Heidelberg cohort of the European Prospective Investigation into Cancer and Nutrition (EPIC), who were 50–75 years at the end of follow-up. Information on diet and lifestyle was collected at recruitment (1994–1998). During a median follow-up of 8.7 years, information on chronic conditions and death were collected.

**Results:**

Overall, the prevalence of multimorbidity (>= 2 concurrent chronic diseases) was 67.3%. Compared to the highest educational category, the lowest was statistically significantly associated with increased odds of multimorbidity in men (OR = 1.43; 95% CI 1.28–1.61) and women (OR = 1.33; 95% CI 1.18–1.57). After adjustment, the positive associations were attenuated (men: OR = 1.28; 95% CI 1.12–1.46; women: OR = 1.16; 95% CI 0.99–1.36). Increasing BMI was more strongly than smoking status an intermediate factor in the association between education and multimorbidity.

**Conclusion:**

In this German population, the prevalence of multimorbidity is high and is significantly associated with educational level. Increasing BMI is the most important predictor of this association. However, even the fully adjusted model, i.e. considering also other known risk factors for chronic diseases, could not entirely explain socio-economic inequalities in multimorbidity. Educational level should be considered in the development and implementation of prevention strategies of multimorbidity.

## Background

Aging populations are characterized by the co-occurrence of multiple chronic conditions. Two or more concurrent chronic conditions are defined as multimorbidity, whereas comorbidity refers to an index disease [1,2]. In the Netherlands, the prevalence of multimorbidity was 33.6% in 50–59 year old men, 60.5% in 60–79 year old men, and 74.2% in men 80 years or older, among women the prevalence was 35.9%, 64.9%, and 79.9%, respectively [2]. Similar numbers were reported from Canada and the U.S. [3,4]. Most studies on multimorbidity were performed in general practice setting [2] or on aggregated population level [5,6].

Socio-economic inequalities were shown to be associated with morbidity and mortality [5-11]. Educational attainment, occupational social class, and income are established indicators of socio-economic position (SEP) [12]. Moreover, pathways through which the socio-economic position influences morbidity have been proposed [13]. For example, SEP differences are likely to reflect differences in smoking [14], diet [15], alcohol consumption and physical activity [16]. Living conditions, chronic stress and access to the health care system were also examined in relation to health disparities [5]. Education as a marker for SEP may be associated with the prevalence of chronic medical conditions in later life. Because multimorbidity has an impact on quality of life and on life expectancy at the individual and population level, the knowledge about modifiable risk factors is valuable [2,17]. Up to now little is known about the determinants and the effect of potential risk factors such as diet and lifestyle on the prevalence of multimorbidity in the general population [17].

Thus, the aims of this study were to investigate the association between educational attainment and multimorbidity and to examine the mediating role of health behaviour among participants aged 50 years or older in the EPIC-subcohort of Heidelberg.

## Methods

### Study Subjects

Between June 1994 and October 1998, 24,540 participants were recruited in the EPIC-Heidelberg cohort, contributing to the European Prospective Investigation into Cancer and Nutrition (EPIC) [18]. Subjects were recruited from the general population [19]. For the present analysis participants younger than 50 years at the end of follow-up (N = 10,759) were excluded. Thus, the analytic cohort comprised 13,781 participants aged between 50 and 75 years at the end of a mean follow-up of 8.7 years.

The baseline assessment included a self–administered food frequency questionnaire (FFQ) and standardized anthropometric measurements including height and weight. Nutrient intake data were calculated from the food consumption data by means of the German Food Composition Tables. The participants were also asked to provide detailed information about their smoking history, physical activity and medical history. The investigation was approved by the ethics committee at the Heidelberg University.

### Follow-up and outcome

Follow-up of the cohort began at the date of recruitment and ended at either death or date of the last contact, whichever came first. Prevalent chronic diseases at recruitment were assessed during face-to-face interview. Incident cases of cancer and other chronic diseases were identified by active follow-up. The follow-up consisted of a questionnaire on physician diagnosed chronic conditions and the year of diagnosis. Chronic diseases were regrouped into cancer (all except non-melanoma skin cancer), diabetes mellitus, thyroid disease, stroke, myocardial infarction (MI), heart diseases (except MI), lung diseases (asthma, chronic obstructive pulmonary disease), gastrointestinal diseases (reflux esophagitis, chronic gastritis, gastric ulcer, Crohn's disease, ulcerative colitis, chronic diverticulitis), liver/kidney diseases, pancreatitis, muscle/bone diseases (rheumatic disease, osteoporosis), diseases of the central nervous system (multiple sclerosis, Alzheimer disease, Parkinson's disease), hypertension, dyslipidemia, and hyperuricemia. All self-reports on incident cases of cancer were verified by comparison with the medical records. However, medical verification of other chronic diseases has not been done, except for a few small validation/pilot studies on incident cases of myocardial infarction, stroke, asthma [20], and diabetes. During follow-up, 800 decedents (576 men and 224 women) were registered, confirmed by death certificates, and all-cause mortality was determined. Participation rate was over 90% in each follow-up round. At the end of the 3^rd ^follow-up, 5.8% of the study participants were lost for follow-up.

The number of concurrent chronic conditions was counted to compute a score of morbidity (0 to ≥ 6). Multimorbidity (yes/no) was defined as two or more concurrent chronic conditions. In addition, multiple metabolic diseases is defined as the concomitant occurrence of metabolic diseases (0, 1, 2, ≥ 3) including diabetes, hypertension, dyslipidemia and hyperuricemia; the dichotomous variable 'metabolic diseases' (yes/no) reflects the existence of any metabolic disease.

### Predictors

Educational attainment was used to characterise a participant's SEP. The highest education achieved was measured by questionnaire at recruitment and summarized according to the German schooling system with three degrees: primary school or less (low), secondary school (middle), and grammar school (high). Subjects without school graduation (N = 113) were assigned to the lowest educational level. The highest category of educational attainment was used as the reference group.

As covariates known or potential risk factors of multimorbidity were considered. Smoking status was categorized as never smoker, ex-smoker or current smoker. Body mass index (BMI) was calculated as weight (kg)/height^2 ^(m^2^). Alcohol consumption (g/day), fruit and vegetable intake (g/day) were included in the models as continuous variables. Physical activity was classified as inactive, moderately inactive, moderately active and active based on occupational activity, cycling, and sports [21].

### Statistical analysis

This is a cross-sectional analysis of multi-morbidity, using all available information on prevalent chronic diseases collected at recruitment of the cohort and during follow-up rounds. Information on lifestyle factors, diet, and anthropometric data (measurements) was collected at recruitment. The prevalence of chronic conditions was analyzed in relation to educational attainment stratified by sex, because the association between educational level and health outcome could vary by sex as former reports have shown [2,3,22]. Age-adjusted logistic regression models were calculated to examine predictors of multimorbidity and Cox proportional hazard models (HR) for mortality, respectively. In order to explore mediating effects we introduced smoking status and increasing BMI (per increments of 5 kg/m^2^) in the age-adjusted models. Adjusted odds ratios (OR_adj_) and 95% confidence intervals (95% CI) for morbidity were calculated by inclusion of age (continuous, year), BMI (continuous, kg/m^2^), fruit intake (continuous, g/day), vegetable intake (continuous, g/day), alcohol consumption (continuous, g/day), smoking habits, and total physical activity in the models. Whether the associations differed by sex or age was assessed by including a cross-product term in the logistic regression equitation, and p for interaction was calculated with the log-likelihood test. For evaluation of the impact of the single multimorbidity scores, multinomial logistic regression models applying the generalized logit function (glogit) in PROC LOGISTIC were calculated. SAS statistical software 9.13 (SAS institute Inc, Cary, NC) was used for all calculations. A p-value < 0.05 was used to determine statistical significance.

## Results

Among 13,781 participants aged 50 to 75 years, the prevalence of multimorbidity was 67.3% in men and 67.4% in women. In men and women, dyslipidemia, hypertension, and gastro-intestinal diseases were the most common chronic conditions. High educational level was related to lower prevalence of dyslipidemia, hypertension, insulin-dependent diabetes, hyperuricemia, myocardial infarction, stroke and cancer in both men and women (Table 1). The distribution of baseline characteristics stratified by educational level and sex are displayed in table 2. Subjects in the highest education category were leaner, whereas in women no educational gradient was found. Irrespective of educational level men were prone to a more sedentary lifestyle.

**Table 1:** Prevalence of chronic conditions among participants aged 50 to 75 years in the in the EPIC-HD cohort by educational level and sex

**Educational level**	**Low**		**Middle**		**High**		**Total**	
**Men**	N	%	N	%	N	%	N	%

Dyslipidemia	2237	58.8	614	57.7	1262	52.2	4113	56.5
Hyperuricemia	708	19.0	140	13.3	275	11.5	1123	15.7
Diabetes mellitus all	643	16.9	129	12.1	241	10.0	1013	13.9
insulin dependent	131	3.4	23	2.2	50	2.1	204	2.8
Thyroid disease	271	7.2	91	8.7	183	7.6	545	7.6
Hypertension	2175	57.1	594	55.8	1188	49.2	3957	54.3
Stroke	204	5.4	41	3.9	78	3.2	323	4.4
Myocardial infarction (MI)	377	9.9	71	6.7	120	5.0	568	7.8
Heart diseases (except MI)	770	21.0	220	21.3	491	20.1	1481	21.0
Lung diseases	261	7.0	80	7.7	169	7.1	510	7.1
Gastrointestinal diseases	1298	34.6	322	30.6	657	27.6	2277	31.7
Pancreatic disease	62	1.7	10	1.0	22	0.9	94	1.3
Liver/kidney diseases	116	3.1	20	1.9	50	2.1	186	2.6
Muscle/bone diseases	607	16.6	88	8.6	206	8.8	901	12.8
Central nervous system	30	0.8	9	0.9	15	0.6	54	0.8
Cancer	575	15.1	146	13.7	277	11.5	998	13.7

**Women**								

Dyslipidemia	1958	56.1	879	52.6	631	47.7	3468	53.5
Hyperuricemia	234	6.8	70	4.3	50	3.8	354	5.6
Diabetes mellitus all	390	11.2	112	6.7	55	4.2	557	8.6
insulin dependent	80	2.3	24	1.4	10	0.8	114	1.8
Thyroid disease	934	27.3	465	28.3	314	24.2	1713	30.0
Hypertension	1916	54.9	799	47.8	544	41.2	3259	50.3
Stroke	119	3.4	33	2.0	21	1.6	173	2.7
Myocardial infarction (MI)	106	3.0	39	2.3	25	1.9	170	2.6
Heart diseases (except MI)	560	16.8	286	17.8	212	16.5	1058	17.0
Lung diseases	279	8.1	126	7.7	104	8.0	509	8.0
Gastrointestinal diseases	1146	33.3	514	31.3	356	27.3	2016	31.6
Pancreatic diseases	46	1.3	21	1.3	18	1.4	85	1.3
Liver/kidney diseases	66	1.9	28	1.7	30	2.3	124	2.0
Muscle/bone diseases	828	25.1	329	20.9	232	18.2	1389	22.6
Central nervous system	20	0.6	8	0.5	7	0.5	35	0.5
Cancer	452	12.9	220	13.2	179	13.5	851	13.1

**Table 2:** Baseline characteristics by educational level and sex of participants aged 50 to 75 years in the EPIC-Heidelberg cohort

	**Men**			**Women**		
**Educational level**	**Low**	**Middle**	**High**	**Low**	**Middle**	**High**

**N**	3810	1065	2418	3492	1670	1322

Multimorbidity^1^: yes (%)	71.0	68.0	61.4	71.0	64.9	61.5
Metabolic disease^2^: yes (%)	81.1	81.3	74.6	78.7	73.4	66.4
Deceased (%)	10.1	6.8	5.0	4.0	2.9	2.8

**Continuous variables**	Median (Q1, Q3)*	Median (Q1, Q3)*	Median (Q1, Q3)*	Median (Q1, Q3)*	Median (Q1, Q3)*	Median (Q1, Q3)*
Age at recruitment (years)	58 (54, 61)	56 (53, 60)	56 (53, 59)	58 (54, 61)	56 (52, 60)	55 (52, 59)
Height (m)	173.30 (169.20, 177.50)	176.00 (171.80, 180.00)	177.00 (172.50, 181.00)	161.10 (157.10, 165.20)	163.60 (159.55, 167.75)	164.90 (161.00, 169.00)
Weight (kg)	83.30 (83.30, 91.40)	82.50 (75.70, 90.50)	81.20 (75.50, 90.00)	70.00 (63.00, 79.05)	67.25 (60.50, 75.60)	65.00 (59.00, 72.00)
Body mass index (kg/m^2^)	27.6 (25.6, 30.0)	26.7 (24.7, 29.1)	25.9 (24.1, 28.1)	26.9 (24.3, 30.3)	25.1 (22.7, 28.0)	23.9 (21.8, 26.6)
Total energy intake (kcal/d)	2036 (1678, 2488)	2006 (1672, 2432)	2072 (1725, 2467)	1603 (1316, 1935)	1608 (1350, 1938)	1677 (1406, 2000)
Fat intake (g/d)	74.10 (57.60, 95.97)	74.67 (58.37, 93.68)	75.06 (59.66, 95.02)	61.77 (48.64, 77.96)	61.81 (48.79, 76.42)	62.49 (50.48, 78.44)
Fruit consumption (g/d)	94.71 (57.64, 136.07)	95.86 (57.97, 147.56)	100.97 (66.16 156.21)	103.42 (81.05, 181.39)	109.20 (85.98, 194.81)	121.45 (91.43, 196.77)
Vegetable consumption (g/d)	99.27 (74.74, 131.09)	103.02 (78.33, 131.53)	108.72 (84.14, 140.74)	106.10 (81.29, 143.60)	110.02 (85.67, 146.44)	122,11 (93.19, 161.12)
Alcohol consumption (g/d)	18.13 (6.93, 39.77)	19.99 (7.80, 39.16)	20.93 (8.89, 38.97)	4.04 (1.06, 11.39)	6.89 (2.18, 17.31)	9.05 (2.80, 21.82)
**Categorical variables**	%	%	%	%	%	%
Smoking status						
Never smokers	29.34	30.99	33.75	63.83	54.37	49.55
Former smokers	48.35	47.79	52.23	20.36	29.46	35.33
Current Smokers	22.31	21.22	14.02	15.81	16.17	15.13
Combined total physical activity index						
Active	8.50	3.10	2.70	9.68	5.95	7.34
Moderately active	42.11	26.57	18.96	62.59	55.88	43.23
Moderately inactive	33.94	30.97	29.69	21.96	25.42	29.70
Inactive	15.44	39.36	48.65	5.76	12.75	19.72

* Q1 Cut point of Quartile 1, Q3 Cut point of Quartile 3

^1^Presence of two or more of the he prevalent chronic conditions mentioned in table 1

^2^Presence of hyperuricemia, diabetes mellitus, hypertension or dyslipidemia

The associations between education and morbidity by sex are shown in table 3. Low education category shows strong gradients with increasing number of chronic diseases overall as well as for metabolic diseases. All relationships were statistically significant, except for metabolic diseases in men.

**Table 3:** Age and multivariate adjusted odds ratios (OR, 95% CI) of the actual number of prevalent chronic diseases by educational level among men and women aged 50 to 75 years in the EPIC-Heidelberg cohort, applying multinomial logistic regression models (Reference category: highest educational level)

	**Men**					**Women**				
**Educational Level**		**Middle**		**Low**			**Middle**		**Low**	
	N	OR	95%-CI	OR	95%-CI	N	OR	95%-CI	OR	95%-CI
**Age-adjusted**										
**Morbidity**^1^										
**0**	657	1				576	1			
**1**	1439	1.35	1.01–1.81	1.08	0.89–1.32	1249	1.31	1.00–1.71	1.41	1.11–1.80
**2**	1595	1.51	1.14–2.02	1.18	0.97–1.44	1458	1.22	0.94–1.59	1.50	1.19–1.91
**3**	1246	1.65	1.22–2.23	1.44	1.16–1.77	1124	1.29	0.98–1.71	1.64	1.28–2.11
**4**	800	1.69	1.21–2.36	1.72	1.37–2.17	634	1.67	1.22–2.31	1.85	1.38–2.48
**5**	374	2.00	1.30–3.09	2.55	1.88–3.45	335	1.77	1.16–2.68	2.33	1.61–3.39
**≥ 6**	293	1.96	1.19–3.23	3.08	2.18–4.33	218	1.54	0.89–2.66	3.31	2.08–5.27
P for trend		<0.001		0.002			0.015		0.003	
**Multivariate-adjusted**^#^										
**Morbidity**^1^										
**0**	647	1				566	1			
**1**	1392	1.32	0.98–1.78	1.02	0.82–1.27	1182	1.26	0.96–1.66	1.31	1.01–1.71
**2**	1511	1.44	1.07–1.93	1.11	0.89–1.38	1344	1.17	0.89–1.53	1.30	1.00–1.68
**3**	1142	1.43	1.04–1.95	1.19	0.94–1.49	1000	1.11	0.82–1.48	1.31	1.00–1.73
**4**	744	1.50	1.06–2.12	1.39	1.07–1.79	551	1.50	1.06–2.12	1.30	0.94–1.81
**5**	353	1.64	1.04–2.57	1.76	1.26–2.45	285	1.47	0.94–2.32	1.60	1.05–2.43
**≥ 6**	253	1.44	0.83–2.49	2.24	1.52–3.29	186	1.47	0.78–2.76	2.53	1.45–4.41
P for trend		0.039		0.003			0.021		0.039	
**Age-adjusted**										
**Metabolic diseases**^2^										
**0**	1504	1				1606	1			
**1**	2480	1.36	1.11–1.66	1.10	0.96–1.27	2562	1.28	1.08–1.53	1.39	1.19–1.63
**2**	2121	1.53	1.24–1.88	1.41	1.22–1.63	1749	1.43	1.17–1.75	1.85	1.55–2.22
**3/4**	1054	1.81	1.39–2.36	2.40	1.99–2.89	454	1.72	1.18–2.50	3.08	2.23–4.26
P for trend		0.009		0.089			0.007		0.042	
**Multivariate-adjusted**^#^										
**Metabolic diseases**^2^										
**0**	1422	1				1483	1			
**1**	2334	1.30	1.06–1.61	0.99	0.85–1.16	2329	1.19	0.99–1.43	1.24	1.04–1.48
**2**	2004	1.36	1.09–1.69	1.16	0.98–1.37	1602	1.29	1.04–1.60	1.38	1.13–1.69
**3/4**	999	1.51	1.14–2.02	1.62	1.31–2.00	402	1.40	0.93–2.12	1.76	1.21–2.55
P for trend		0.043		0.113			0.012		0.018	

^# ^adjusted for smoking status, BMI (continuous; kg/m2), fruit intake (continuous; g/day), vegetable intake (continuous; g/day), alcohol consumption (continuous; g/day), age (continuous; years), combined total physical activity index (inactive, moderately inactive, moderately active, active)

^1^Number of the prevalent chronic conditions mentioned in table 1

^2^Number of prevalent metabolic diseases, including hyperuricemia, diabetes mellitus, hypertension and dyslipidemia

In the adjusted models, low educational attainment of men was statistically significantly associated with an increased prevalence of most chronic diseases except for cancer and stroke in men (data not shown). Among women no associations were found for cancer, myocardial infarction, pancreatic disease, liver/kidney diseases and thyroid disease. Diseases of the heart (other than MI), lung, liver/kidney, and central nervous system were not associated with educational level in men and women (data not shown).

Table 4 displays the age- and multivariate adjusted odds ratios for multimorbidity by education and sex. In the age-adjusted models, the associations between educational level and multimorbidity were slightly stronger in men (middle vs. high level OR = 1.32; 95% CI 1.12–1.55 and low vs. high level OR = 1.43; 95% CI 1.28–1.61) than in women (middle vs. high level OR = 1.13; 95% CI 0.97–1.33 and low vs. high level OR = 1.33; 95% CI 1.18–1.57). In women, the educational level had stronger impact on the occurrence of metabolic diseases (low vs. high level OR = 1.64; 95% CI 1.42–1.90) than in men (low vs. high level OR = 1.38; 95% CI 1.22–1.57). However, none of these suggested statistical interactions between sex and education were of statistical significance (multimorbidity p = 0.670 and metabolic disease p = 0.447). Differences in the strength of the association may be predominantly attributed to diabetes, which was more strongly associated with education in women than in men. By adjustment for potential risk factors the associations for multimorbidity, metabolic disease and mortality were attenuated. Concerning all-cause mortality, the introduction of smoking status and BMI in age-adjusted models attenuated the estimates with higher mortality among subjects in the lowest educational category.

**Table 4:** Age and multivariate adjusted odds (OR, 95% CI) and hazard ratios (HR, 95% CI) of multimorbidity, metabolic diseases, and mortality, including additional models adjusted for smoking status or BMI, by educational level among men and women aged 50 to 75 years in the EPIC-Heidelberg cohort

	**Men**						**Women**				
**Educational level**	**High**		**Middle**		**Low**		**High**	**Middle**		**Low**	
	OR		OR	95%-CI	OR	95%-CI		OR	95%-CI	OR	95%-CI

**Multimorbidity: yes**^1^											
Age-adjusted		1	1.32	1.12–1.55	1.43	1.28–1.61	1	1.13	0.97–1.33	1.33	1.18–1.57
Smoking status -adjusted *		1	1.34	1.14–1.58	1.46	1.30–1.64	1	1.14	0.97–1.34	1.38	1.20–1.60
BMI- adjusted **		1	1.23	1.05–1.45	1.24	1.10–1.40	1	1.05	0.89–1.23	1.12	0.96–1.30
Fully adjusted #		1	1.21	1.02–1.43	1.28	1.12–1.46	1	1.06	0.89–1.25	1.16	0.99–1.36
**Metabolic diseases: yes**^2^											
Age-adjusted		1	1.48	1.23–1.77	1.38	1.22–1.57	1	1.35	1.15–1.59	1.64	1.42–1.90
Smoking status -adjusted *		1	1.51	1.26–1.81	1.41	1.25–1.61	1	1.36	1.16–1.60	1.67	1.44–1.93
BMI- adjusted **		1	1.34	1.11–1.61	1.09	0.96–1.25	1	1.21	1.03–1.42	1.25	1.18–1.46
Fully adjusted #		1	1.37	1.13–1.66	1.15	1.00–1.33	1	1.24	1.04–1.47	1.33	1.13–1.56
	HR		HR	95%-CI	HR	95%-CI		HR	95%-CI	HR	95%-CI
**All-cause mortality: yes**											
Age-adjusted		1	1.38	1.02–1.85	1.90	1.54–2.34	1	1.04	0.67–1.61	1.30	0.89–1.90
Smoking status -adjusted *		1	1.26	0.94–1.69	1.69	1.37–2.09	1	1.05	0.68–1.62	1.33	0.92–1.95
BMI- adjusted **		1	1.36	1.01–1.83	1.82	1.46–2.25	1	0.99	0.64–1.54	1.23	0.83–1.81
Fully adjusted #		1	1.26	0.91–1.74	1.60	1.25–2.05	1	1.32	0.78–2.22	1.49	0.92–2.41

* adjusted for smoking status and age (continuous; years)

** adjusted for BMI (continuous; kg/m^2^), and age (continuous; years)

# adjusted for smoking status, BMI (continuous; kg/m^2^), fruit intake (continuous; g/day), vegetable intake (continuous; g/day), alcohol consumption (continuous; g/day), age (continuous; years), combined total physical activity index (inactive, moderately inactive, moderately active, active)

^1^Presence of two or more of the prevalent chronic conditions mentioned in table 1

^2^Presence of prevalent metabolic diseases, including hyperuricemia, diabetes mellitus, hypertension or dyslipidemia

The introduction of BMI, but not smoking, in the age-adjusted models attenuated the estimates for multimorbidity and metabolic disease indicating that increasing BMI may contribute as intermediate factor to the relationship with education. Smoking habits had a moderate effect on the association between education and mortality in men, while increasing BMI had little effect on this relationship. In women, smoking status and increasing BMI had little impact on the relationship between education and mortality. Further adjustment for potential confounders such as alcohol consumption, fruit and vegetable intake, and physical activity did not substantially influence the associations between education and multimorbidity in both men and women.

Table 5 shows the results of analyses stratified by age group. In participants aged ≤ 60 years at recruitment, lower educational level was associated with higher prevalence of multimorbidity as well as for multiple metabolic diseases in men and in women. However, only in men the statistical interactions between age and education were significant for multimorbidity (p = 0.008) and metabolic diseases (p = 0.008) but not in women (p = 0.808 and p = 0.647).

**Table 5:** Multivariate adjusted odds ratios (OR, 95% CI) of multimorbidity and metabolic diseases by educational level and sex according to age groups among participants aged 50 to 75 years in the EPIC-Heidelberg cohort

	**Men**					**Women**				
**Educational Level**	**High**	**Middle**		**Low**		**High**	**Middle**		**Low**	
	OR	OR	95%-CI	OR	95%-CI	OR	OR	95%-CI	OR	95%-CI
**Multimorbidity: yes**^1^										
Age <= 60 years at recruitment ^#^	1	1.24	1.03–1.50	1.36	1.17–1.59	1	1.05	0.87–1.26	1.20	1.00–1.43
Age > 60 years at recruitment ^#^	1	0.95	0.65–1.39	0.90	0.68–1.19	1	1.08	0.70–1.67	0.93	0.64–1.35
**Metabolic diseases: yes**^2^										
Age <= 60 years at recruitment ^#^	1	1.38	1.12–1.71	1.18	1.00–1.38	1	1.24	1.03–1.49	1.32	1.11–1.58
Age > 60 years at recruitment^#^	1	1.16	0.74–1.83	0.97	0.71–1.32	1	1.16	0.74–1.82	1.24	0.84–1.83

^§ ^adjusted for BMI (continuous; kg/m2), fruit intake (continuous; g/day), vegetable intake (continuous, g/day), alcohol consumption (continuous; g/day), age (continuous; years, total physical activity (inactive, moderately inactive, moderately active, active)

^≠ ^adjusted for smoking status, fruit intake (continuous; g/day), vegetable intake (continuous; g/day), alcohol consumption (continuous; g/day), age (continuous years), combined total physical activity index (inactive, moderately inactive, moderately active, active)

^# ^adjusted for smoking status, BMI (continuous; kg/m2), fruit intake (continuous; g/day), vegetable intake (continuous; g/day), alcohol consumption (continuous; g/day), age (continuous years), combined total physical activity index (inactive, moderately inactive, moderately active, active)

^1^Presence of two or more of the prevalent chronic conditions mentioned in table 1

^2^Presence of hyperuricemia, diabetes mellitus, hypertension or dyslipidemia

## Discussion

Overall, multimorbidity was more prevalent among men and women aged 50–75 years with low educational level than in subjects with higher educational level. Higher BMI, but not smoking status, was identified as an intermediate factor in the relationships between education and multimorbidity and metabolic disease. However, the adjustment for these risk factors did not completely explain the effect of education on health outcomes.

### Chronic diseases

Consistent with our findings, other authors identified hypertension, dyslipidemia, chronic ischemic heart disease, back pain and diabetes as most prevalent diagnoses among 60 to 70 year old persons in Germany [23]. The higher prevalence of gastrointestinal diseases may be attributed to younger age of the participants in our study. The prevalence of central-nervous system diseases, however, is likely to be underestimated because of the increasing study participants' disability to complete questionnaires.

Our observations are consistent with the well-known paradox that mortality in women is lower than in men of the same age despite higher levels of morbidity among women [24]. In our data, the sex difference of mortality was weaker compared to a previous review [25]. This may be explained by increasing smoking prevalence among women during the past decades [26].

### Intermediaries

Differences in socio-economic position are associated with inequalities in health that may be mediated by differences in health-related behaviour [27]. In line with former research [15,27-30], we observed that participants with a higher educational level were leaner, ate more fruits and vegetables, and were less likely to be smokers at recruitment. Consistent with our results, it was also noted that moderate drinking was not associated with a SEP gradient, while heavy drinking was more prevalent at lower levels [31,32]. We observed that the smoking prevalence and, in consequence, number of smoking related diseases were higher in low education groups than in their counterparts. This observation is in consistency with other reports [28,31]. In our study, however, middle and highly educated men were less physically active than less educated men (please see table 2). The combination of physical inactivity and other adverse lifestyle factors could explain the high prevalence of metabolic disease in men at intermediate educational level.

Our observation that low SEP was recognized as a determinant for higher risk of chronic diseases are in line with the literature [5,6,31]. We observed that the association of education differed by disease and adjustment attenuated the relationships differentially [6,33]. Our results suggest that higher BMI, but not smoking status, may act as an intermediate factor of education on multimorbidity. However, smoking status attenuated the estimates for mortality. Potential explanation could be that in the age group 50 to 75 years smoking habits are rather associated with mortality than with multimorbidity.

Research on mortality has shown that the associations with education were partially eliminated after adjustment for covariates [34]. Other authors found that both social class in childhood and adulthood contribute to disease risk applying objective measures in mid-life [35].

### Multimorbidity

Consistent with other studies, we observed that low educational level was statistically significantly associated with a higher prevalence of multimorbidity compared to high educational level [2,6]. Stronger associations between education and multimorbidity were found among the participants aged 50 to 60 years than among elders, which can be explained by premature mortality in low SEP groups [36]. Growing evidence exists that SEP differences are also reflected in biological markers of disease [5,6]. In a cohort study on cardiovascular risk factors among men in France and Northern Ireland, education was inversely associated with blood pressure and BMI but not with total cholesterol concentration [31]. Our data further suggest that the educational attainment is not only associated with multimorbidity, but that the social gradient is related to the number of diseases. Our observation that education has an impact on multimorbidity is supported by numerous studies on the relationship between low education and high mortality [9,13,23]. Further evidence comes from a study on morbidity during the last years of life among US citizens, which revealed lower morbidity and disability in those with higher education [37].

Further unmeasured factors may contribute to the relationship between education and multimorbidity such as psychosocial factors, stress and other environmental factors [7,38-40]. It has been hypothesized that low control in terms of autonomy and low social participation are likely contributors to health inequalities [41]. Psychosocial variables such as employment, active coping style, high occupational class and an external locus of control were associated with low prevalence of multimorbidity [42].

Sex differences in health were hypothesized to be related to differential exposure and differential vulnerability [43]. In the present study, pancreatitis and hyperuricemia were more frequent in men and stroke was more frequent in women. Higher prevalence of diabetes in women than in men was found by other authors [6,44]. Our results suggest differential associations between education and multimorbidity by sex, which may be explained by differences in lifestyle [26], however, these differences could also be due to chance variation by stratification. Also behavioural aspects are likely to be linked with the outcome [40,43].

Some potential limitations of this study should be considered. We analyzed school attainment as indicator of SEP, but no other indicator such as profession or income. However, measures for adulthood SEP such as occupation or income and measures of economic distress have been shown to be in good correlation with each other [45]. Participants of EPIC-Heidelberg were recruited from the general population [19], but the response rate in less educated subjects tends to be lower than in well-educated ones, implying that the real differences may actually be underestimated.

Our study was largely based on the participants' self-reports, which could have introduced some bias. However, all self-reports on cancer were verified by comparison with medical records, and validation studies were conducted for diabetes, myocardial infarction, and stroke. Misreporting could be linked to the respondents' educational levels, resulting in underestimation of educational inequalities in a previous study [46]. Also, non-response is higher in groups of low SEP and of ill-health [47], which may also result in attenuated estimates. Information on habitual diet and lifestyle factors may be affected by morbidity itself since reverse causation can not be excluded. However, dietary habits and other lifestyle factors were found to be relatively stable over time in our cohort [48]. For some chronic diseases it is difficult to determine whether or not they can be considered independently from each other, such as diabetes mellitus, hypertension, and myocardial infarction [49]. From the public health point-of-view, however, this is irrelevant because persons with several diseases are likely to take more medications and to have more medical consultations.

The strengths of this study are the use of prospective data, its population-based approach, and the possibility to adjust for relevant confounding variables.

## Conclusion

In this large prospective study, low educational attainment was associated with higher prevalence of multimorbidity. Our results suggest that higher BMI is an intermediate factor for multimorbidity among subjects aged 50 to 75 years. Adjustment for other potential risk factors did not completely explain the relationships. Aspects of SEP, sex and BMI need consideration in public health actions to prevent multimorbidity.

## Competing interests

The authors declare that they have no competing interests.

## Authors' contributions

GN and RP initiated and designed the project and GN conducted the statistical analyses. JL is principal investigator of the EPIC-Heidelberg cohort study and contributed to the study design. SR and SH were involved in data collection and contributed to the variable selection. SB and SR provided advice on design and statistics. All authors provided specialist knowledge in the advancement of the analyses and provided input in re-drafting the manuscript. GN drafted the first manuscript and wrote the final version, which was seen and approved by all authors.

## Pre-publication history

The pre-publication history for this paper can be accessed here:


